# iPoLNG—An unsupervised model for the integrative analysis of single-cell multiomics data

**DOI:** 10.3389/fgene.2023.998504

**Published:** 2023-02-07

**Authors:** Wenyu Zhang, Zhixiang Lin

**Affiliations:** Department of Statistics, The Chinese University of Hong Kong, Hong Kong, China

**Keywords:** integrative analysis, single-cell multi-omics data, probabilistic non-negative matrix factorization, generative model, unsupervised learning, stochastic variational inference

## Abstract

Single-cell multiomics technologies, where the transcriptomic and epigenomic profiles are simultaneously measured in the same set of single cells, pose significant challenges for effective integrative analysis. Here, we propose an unsupervised generative model, iPoLNG, for the effective and scalable integration of single-cell multiomics data. iPoLNG reconstructs low-dimensional representations of the cells and features using computationally efficient stochastic variational inference by modelling the discrete counts in single-cell multiomics data with latent factors. The low-dimensional representation of cells enables the identification of distinct cell types, and the feature by factor loading matrices help characterize cell-type specific markers and provide rich biological insights on the functional pathway enrichment analysis. iPoLNG is also able to handle the setting of partial information where certain modality of the cells is missing. Taking advantage of GPU and probabilistic programming, iPoLNG is scalable to large datasets and it takes less than 15 min to implement on datasets with 20,000 cells.

## 1 Introduction

With the rapid development of single-cell technologies, the abundant biological information in the single cell is collected at unprecedented resolution. More recently, sequencing methods enable the simultaneous measurement of epigenome and transcriptome from a common set of single cells. For example, sci-CAR (Cao et al., 2018) jointly profiles chromatin accessibility and mRNA (CAR) in each of thousands of single cells; SNARE-seq (Chen et al., 2019b), SHARE-seq (Ma et al., 2020), Paired-seq (Zhu et al., 2019) can measure chromatin accessibility and gene expression in the same single cell; Paired-Tag (Zhu et al., 2021) is an ultra-high-throughput method for joint profiling of histone modifications and transcriptome in single cells to produce cell-type-resolved maps of chromatin state and transcriptome in complex tissues.

The single-cell multiomics datasets generated by these technologies pose challenges for effective integrative analysis due to the characteristics of the datasets. First, the single-cell data is high-dimensional yet very sparse, and high technical variation is present in single-cell datasets. Second, the level of noise in chromatin accessibility data or histone modification data is usually higher than gene expression data in single-cell multiomics datasets, which suggests that different data modalities cannot be simply treated the same.

Computational tools for the integrative analysis of single-cell assays are essential to provide more comprehensive biological insights at the cellular level. Integration problems in single-cell biology can be divided into those associated with the integration of unmatched data (that is, different modalities profiled from different cells) or matched (that is, different modalities profiled from the same cell) data (Miao et al., 2021). A few methods have been developed for the integrative analysis of unmatched data (Duren et al., 2018; Zeng et al., 2019; Cao et al., 2020; Lin et al., 2020; Wangwu et al., 2021; Zeng et al., 2021; Zeng and Lin, 2021; Cao et al., 2022; Demetci et al., 2022), which are not applicable to matched data. Some matched data integration methods (Kim et al., 2020; Wang et al., 2020; Gayoso et al., 2021) are designed for technologies that jointly profile transcriptomic and surface protein data such as CITE-seq (Stoeckius et al., 2017) and REAP-seq (Peterson et al., 2017). In this study, we mainly focus on single-cell multiomics technologies simultaneously measuring transcriptomic and epigenomic profiles in the same individual cells. Unsupervised methods have been developed for this type of data, including Multi-Omics Factor Analysis (MOFA+) (Argelaguet et al., 2018; Argelaguet et al. 2020), single-cell Aggregation and Integration (scAI) (Jin et al., 2020) and jointly semi-orthogonal non-negative matrix factorization (JSNMF) (Ma et al., 2022). Both MOFA+ and scAI infers a low-dimensional representation of the data using a small number of latent factors that are expected to capture the heterogeneous cellular variability. The key difference between MOFA+ and scAI is that MOFA+ is based on the Bayesian Group Factor Analysis framework, while scAI is based on non-negative matrix factorization. JSNMF assumes different latent variables for the two molecular modalities, and integrates the information of transcriptomic and epigenomic data with consensus graph fusion.

In this paper, we propose an unsupervised generative model, iPoLNG, for the effective and scalable integration of single-cell multiomics data, where transcriptomic and epigenomic (chromatin accessibility or histone modifications) data were obtained from the same cell. iPoLNG reconstructs low-dimensional representations of the cells and features using computationally efficient stochastic variational inference by modelling the discrete counts in single-cell multiomics data with latent factors. The hyperparameters introduced to tackle the difference in the levels of noise across different data modalities can be estimated automatically through a heuristic procedure. By applying iPoLNG to real datasets, we demonstrate that the low-dimensional representation of cells leads to improved clustering performance, and the feature by factor loading matrices help characterize cell-type specific markers and provide rich and consistent biological insights on the functional pathway enrichment analysis. iPoLNG is also able to handle the setting of partial information where certain modality of the cells is missing. We also illustrate the effectiveness of our model in the simulation study. Taking advantage of GPU and probabilistic programming, iPoLNG is scalable to large datasets and it takes less than 15 min to implement on datasets with 20,000 cells.

## 2 Materials and methods

### 2.1 PoLNG for one data modality

We first introduce some notations. Let $W\in N_{I\times J}$ denote the cell by feature count data for one single-cell data modality, *I* the number of cells, *J* the number of features, $R^{*}$ the notation for non-negative real numbers and *K* the number of latent factors, which is much smaller than *I* or *J*.

#### 2.1.1 Model formulation

The basic idea of the PoLNG model is to model the data matrix ***W*** as random variables sampled from Poisson distributions, the parameters of which are determined by two low-rank non-negative matrices $L\in R_{I\times K}^{*}$ sampled from Gamma distributions and $\Theta \in R_{K\times J}^{*}$ . ***L*** can be viewed as the low-dimensional representation of the cells, while **Θ** can be viewed as the loading matrix for the features. More specifically, the formulation of the model is proposed as follows:

$$\begin{array}{c} l_{i,k}∼Gamma\left(\alpha_{i,k},\beta_{i,k}\right),\\ \theta_{k,\cdot }=\sigma \left({\tilde{\theta }}_{k,\cdot }\right),{\tilde{\theta }}_{k,\cdot }∼Logit-Normal\left(\mu_{k},\Sigma_{k}\right),\\ w_{i,j}∼Poisson\left(s_{i}\overset{K}{\underset{k=1}{\sum }}l_{i,k}\theta_{k,j}\right), \end{array}$$ (1)

where *σ*(⋅) is the softmax function, the *l*th element of which is given by

$$\sigma_{l}\left({\tilde{\theta }}_{k,\cdot }\right)=\frac{e^{{\tilde{\theta }}_{k,l}}}{\sum_{j=1}^{J}e^{{\tilde{\theta }}_{k,j}}},$$ (2)

***μ***_*k*_ is a vector of length *J* serving as the mean of the Logit-Normal distribution, **Σ**_*k*_ is a *J* by *J* diagonal matrix serving as the covariance matrix of the Logit-Normal distribution, and *s*_*i*_ is the scaling factor to take into account the sequencing depth for the *i*th cell.

The PoLNG model is designed to facilitate the downstream analysis of single-cell data. In general, each column of ***L*** represents a latent factor that can disentangle the heterogeneous cellular information, while each row of **Θ** represents a latent factor for features. Since **Θ** is constrained to have row sum equal to 1, we also impose a soft normalization on ***L*** by introducing the scaling factor *s*_*i*_.

We further illustrate the choice of *s*_*i*_. Utilizing the simplex constraint for each row of **Θ**, we have

$$E\left(\overset{J}{\underset{j=1}{\sum }}w_{i,j}|l_{i,\cdot }\right)=\overset{J}{\underset{j=1}{\sum }}E\left(w_{i,j}|l_{i,\cdot }\right)=\overset{J}{\underset{j=1}{\sum }}s_{i}\overset{K}{\underset{k=1}{\sum }}l_{i,k}\theta_{k,j}=s_{i}\overset{K}{\underset{k=1}{\sum }}l_{i,k}\overset{J}{\underset{j=1}{\sum }}\theta_{k,j}=s_{i}\overset{K}{\underset{k=1}{\sum }}l_{i,k},$$ (3)

which suggests that the choice of *s*_*i*_ will softly constrain the row sum of ***L***. To alleviate the effect of the difference in sequencing depth for the cells, we constrain the summation $\sum_{k=1}^{K}l_{i,k}$ to be around 1, and set *s*_*i*_ as

$$s_{i}=\overset{J}{\underset{j=1}{\sum }}w_{i,j}.$$ (4)

To obtain the parameter estimation, we implement the stochastic variational inference (SVI) algorithm (Hoffman et al., 2013) with the deep universal probabilistic program Pyro (Bingham et al., 2019). Conditional on the data ***W***, we assume the independency across all *l*_*i*,*k*_, across all ${\tilde{\theta }}_{k,\cdot }$ , and between ***L*** and **Θ**. The variational distributions are set as

$$\begin{array}{c} l_{i,k}|W∼Gamma\left(a_{i,k},b_{i,k}\right),\\ \theta_{k,\cdot }=\sigma \left({\tilde{\theta }}_{k,\cdot }\right),{\tilde{\theta }}_{k,\cdot }|W∼Logit-Normal\left({\bar{\mu }}_{k},{\bar{\Sigma }}_{k}\right). \end{array}$$ (5)

By default, the hyperparameters in the prior in model (1) are set as

$$\alpha_{i,k}=0.1,\beta_{i,k}=K\alpha_{i,k},\mu_{k}=0,\Sigma_{k}=I\text{ for all }i,k.$$ (6)

The default initial values for the parameters in the variational distributions are set as

$$a_{i,k}=b_{i,k}=0.5,{\bar{\mu }}_{k}=0,{\bar{\Sigma }}_{k}=0.1I\text{ for all }i,k.$$ (7)

The estimated parameters $\hat{L}$ and $\hat{\Theta }$ are computed as the mode of the corresponding variational distributions:

$${\hat{l}}_{i,k}=\frac{{\hat{a}}_{i,k}-1}{{\hat{b}}_{i,k}}I\left({\hat{a}}_{i,k}>1\right),\;{\hat{\theta }}_{k,\cdot }=\sigma \left({\hat{\bar{\mu }}}_{k}\right).$$ (8)

Note that the covariance matrix **Σ**_*k*_ in the Logit-Normal distribution can capture the correlation structure in the features if we do not constrain it to be diagonal. However, if we do not impose the diagonal constraint on the covariance matrix, the number of free parameters in one covariance matrix will increase from *J* to *J*(*J* + 1)/2, which brings high computational cost. Therefore, we assume that the covariance matrix **Σ**_*k*_ is diagonal for efficient and lightweight implementation of the model.

#### 2.1.2 Relationship to existing models

The PoLNG model can be considered as a special case in Poisson Factor Analysis (Zhou et al., 2012) under novel priors. One model that is closely related to our PoLNG model is called the Gamma-Poisson (GaP) model (Canny, 2004), and Buntine and Jakulin (2006) extended the GaP model with Dirichlet priors on ***θ***_*k*,⋅_:

$$l_{i,k}∼Gamma\left(\alpha_{i,k},\beta_{i,k}\right),\theta_{k,\cdot }∼Dirichlet\left(\gamma_{k}\right),w_{i,j}∼Poisson\left(\overset{K}{\underset{k=1}{\sum }}l_{i,k}\theta_{k,j}\right),$$ (9)

where ***γ***_*k*_ is the concentration parameter in the Dirichlet distribution.

The difference between the GaP model and the PoLNG model is that the Dirichlet prior in the GaP model is replaced by the Logit-Normal distribution in the softmax basis in the PoLNG model, as suggested by Atchison and Shen (1980). This change keeps the simplex constraint for each row of **Θ**, but it allows for carrying out unconstrained optimization of the cost function without the simplex constraints (Srivastava and Sutton, 2017). Moreover, it improves computational stability in the stochastic variational inference method. When coded in the Pyro program, our model (1) with logit-normal distribution is less likely to raise numerical errors than model (9) with the Dirichlet prior.

For the parameter estimation in the GaP model, Buntine and Jakulin (2006) proposed a mean-field variational inference algorithm and a Gibbs sampling algorithm by introducing a latent variable with dimension *I* × *J* × *K*. However, because *I* and *J* are typically large in single-cell data, the computational and memory cost of introducing such a 3-dimensional latent variable would be unaffordable for a moderate *K*. In contrast, our SVI algorithm does not introduce memory consuming latent variables and enables GPU acceleration when coded in the deep universal probabilistic program Pyro.

The PoLNG model is also related to non-negative matrix factorization (NMF) (Lee and Seung, 1999). It can be viewed as a probabilistic non-negative matrix factorization model, as it models the expectation of the count data as the multiplication of two non-negative matrices, i.e., $E\left(W\right)=L^{*}\Theta$ , where ***L*** * = ***SL*** and ***S*** is an *I* by *I* diagonal matrix with diagonal elements {*s*_1_, *s*_2_, *…* , *s*_*I*_}. To alleviate the model identification problem, the prior on **Θ** ensures each row of **Θ** is normalized to have sum 1, thus avoiding the case where $\left(\tilde{L},\tilde{\Theta }\right)=\left(aL,\frac{1}{a}\Theta \right)$ is also a possible solution for any *a* > 0, *a* ≠ 1. However, this kind of topic models also typically suffer from the label switching problem. For example, if we impose identical priors to all the components in ***L*** and **Θ**, switch the *k*_1_-th and *k*_2_-th columns in ***L*** and switch the *k*_1_-th and *k*_2_-th rows in **Θ** at the same time, then we obtain another solution that leads to the same data likelihood or evidence lower bound (ELBO) in variational inference. But we need not worry about this label switching problem as the switching of factor indices has little influence on the downstream analysis.

### 2.2 iPoLNG for multiomics data

For the single-cell multiomics data, suppose we have two data modalities, $W^{\left(m\right)}\in N_{I\times J_{m}}$ for *m* = 1, 2. Both data modalities measure the information for the same set of *I* cells, but they represent different types of genomic features. For example, ***W*** ^(1)^ can be gene expression data, the features being genes, and ***W*** ^(2)^ can be chromatin accessibility data, the features being peaks.

**FIGURE 1:**
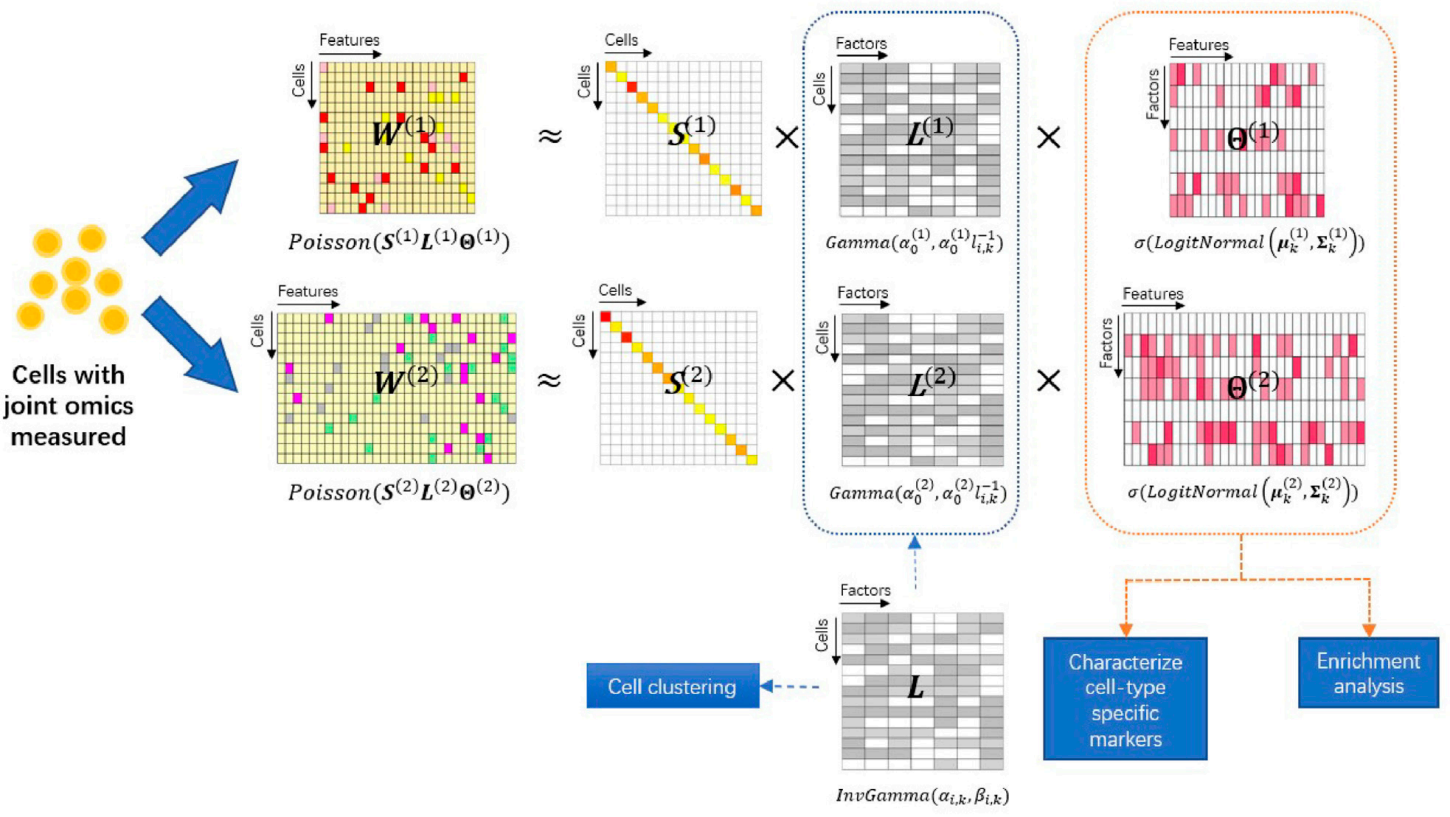
Overview of the iPoLNG model. The input consists of two data modalities measuring different aspects of biological profiles in the same set of cells. Each data modality ***W*** ^(*m*)^ is approximated by the matrix product of a diagonal matrix ***S*** ^(*m*)^ that takes into account the cell sequencing depth, a feature loading matrix **Θ** ^(*m*)^ and a cell loading matrix ***L*** ^(*m*)^ for *m* = 1, 2. The feature loading matrices can characterize cell-type specific markers and facilitate functional pathway enrichment analysis. The cell loading matrices have different variances due to the levels of noise across different data modalities, but share the same mean, ***L***, which represents the low-dimensional cell embedding and facilitates cell clustering.

To model single-cell multiomics data, we extend the PoLNG model to the iPoLNG model. The model overview is presented in Figure 1. In the iPoLNG model, we model the expectation of the *m*th data modality as the multiplication of two non-negative matrices, i.e., $E\left(W^{\left(m\right)}\right)=L^{*\left(m\right)}\Theta^{\left(m\right)}$ , where ***L*** *^(*m*)^ = ***S*** ^(*m*)^ ***L*** ^(*m*)^ and ***S*** ^(*m*)^ is an *I* by *I* diagonal matrix that takes into account the sequencing depth for the cells in the *m*th data modality. Then we link all ***L*** ^(*m*)^ to a common non-negative matrix ***L***, each element of which follows an inverse gamma distribution.

More specifically, the iPoLNG model is proposed as follows:

$$\begin{array}{c} l_{i,k}∼InverseGamma\left(\alpha_{i,k},\beta_{i,k}\right),\\ l_{i,k}^{\left(m\right)}|l_{i,k}∼Gamma\left(\alpha_{0}^{\left(m\right)},\alpha_{0}^{\left(m\right)}l_{i,k}^{-1}\right)\text{ for }m=1,2,\\ \theta_{k,\cdot }^{\left(m\right)}=\sigma \left({\tilde{\theta }}_{k,\cdot }^{\left(m\right)}\right),{\tilde{\theta }}_{k,\cdot }^{\left(m\right)}∼Logit-Normal\left(\mu_{k}^{\left(m\right)},\Sigma_{k}^{\left(m\right)}\right)\text{ for }m=1,2,\\ w_{i,j}^{\left(m\right)}∼Poisson\left(s_{i}^{\left(m\right)}\overset{K}{\underset{k=1}{\sum }}l_{i,k}^{\left(m\right)}\theta_{k,j}^{\left(m\right)}\right)\text{ for }m=1,2, \end{array}$$ (10)

where *l*_*i*,*k*_ is the element in the *i*th row and the *k*th column in ***L***, *α*_*i*,*k*_, *β*_*i*,*k*_ is the shape and scale parameter in the inverse Gamma distribution, $l_{i,k}^{\left(m\right)}$ is the element in the *i*th row and the *k*th column in ***L*** ^(*m*)^, $\alpha_{0}^{\left(m\right)}$ is the hyperparameter that tackles the level of noise in the *m*th data modality, $\theta_{k,\cdot }^{\left(m\right)}$ is the *k*th row vector in **Θ** ^(*m*)^, $\mu_{k}^{\left(m\right)}$ is a vector of length *J*_*m*_ serving as the mean of the Logit-Normal distribution, $\Sigma_{k}^{\left(m\right)}$ is a *J*_*m*_ by *J*_*m*_ diagonal matrix serving as the covariance matrix of the Logit-Normal distribution, $w_{i,j}^{\left(m\right)}$ is the element in the *i*th row and the *j*th column in ***W*** ^(*m*)^, and $s_{i}^{\left(m\right)}$ is the scaling factor that accounts for the sequencing depth for each cell in the *m*th data modality.

In the iPoLNG model, we use an inverse gamma distribution to model the elements in ***L***, such that $l_{i,k}^{-1}$ follows a gamma distribution, based on the fact that gamma distribution is the conjugate prior to the gamma distribution with a known shape parameter. To tackle different levels of noise across the data modalities, we assume that the expectations of $l_{i,k}^{\left(m\right)}$ given *l*_*i*,*k*_ are identical for all *m*, but the variances vary according to the hyperparameter $\alpha_{0}^{\left(m\right)}$ :

$$E\left(l_{i,k}^{\left(m\right)}|l_{i,k}\right)=l_{i,k},var\left(l_{i,k}^{\left(m\right)}|l_{i,k}\right)=\frac{l_{i,k}^{2}}{\alpha_{0}^{\left(m\right)}}.$$ (11)

note that the variance of $l_{i,k}^{\left(m\right)}$ given *l*_*i*,*k*_ will decrease when $\alpha_{0}^{\left(m\right)}$ increases. When $\alpha_{0}^{\left(m\right)}$ is large, $l_{i,k}^{\left(m\right)}$ will tend to be close to *l*_*i*,*k*_, which indicates that the level of noise in the *m*th data modality is low.

$s_{i}^{\left(m\right)}$ is set the same way as that in the PoLNG model:

$$s_{i}^{\left(m\right)}=\overset{J_{m}}{\underset{j=1}{\sum }}w_{i,j}^{\left(m\right)}.$$ (12)

To obtain the parameter estimation in Pyro, conditional on the data ***W*** ^(1)^, ***W*** ^(2)^, we assume the independency across all $l_{i,k},l_{i,k}^{\left(1\right)},l_{i,k}^{\left(2\right)}$ , across all ${\tilde{\theta }}_{k,\cdot }^{\left(1\right)},{\tilde{\theta }}_{k,\cdot }^{\left(2\right)}$ , and among ***L***, ***L*** ^(1)^, ***L*** ^(2)^, **Θ** ^(1)^, **Θ** ^(2)^. The variational distributions are set as

$$\begin{array}{c} l_{i,k}|W^{\left(1\right)},W^{\left(2\right)}∼InverseGamma\left(a_{i,k},b_{i,k}\right),\\ l_{i,k}^{\left(m\right)}|W^{\left(1\right)},W^{\left(2\right)}∼Gamma\left(a_{i,k}^{\left(m\right)},b_{i,k}^{\left(m\right)}\right)\text{ for }m=1,2,\\ \theta_{k,\cdot }^{\left(m\right)}=\sigma \left({\tilde{\theta }}_{k,\cdot }^{\left(m\right)}\right),{\tilde{\theta }}_{k,\cdot }^{\left(m\right)}|W^{\left(1\right)},W^{\left(2\right)}∼Logit-Normal\left({\bar{\mu }}_{k}^{\left(m\right)},{\bar{\Sigma }}_{k}^{\left(m\right)}\right)\text{ for }m=1,2. \end{array}$$ (13)

by default, the hyperparameters in the prior in model (10) are set as

$$\alpha_{i,k}=1,\beta_{i,k}=\left(\alpha_{i,k}+1\right)/K,\mu_{k}^{\left(m\right)}=0,\Sigma_{k}^{\left(m\right)}=I\text{ for all }i,k,m.$$ (14)

if no initial values are provided, the default initial values for the parameters in the variational distributions are set as

$$a_{i,k}=b_{i,k}=a_{i,k}^{\left(m\right)}=b_{i,k}^{\left(m\right)}=0.5,{\bar{\mu }}_{k}^{\left(m\right)}=0,{\bar{\Sigma }}_{k}^{\left(m\right)}=0.1I\text{ for all }i,k,m.$$ (15)

the estimated parameters $\hat{L},{\hat{L}}^{\left(m\right)}$ and ${\hat{\Theta }}^{\left(m\right)}$ are computed as the mode of the corresponding variational distributions:

$${\hat{l}}_{i,k}=\frac{{\hat{b}}_{i,k}}{{\hat{a}}_{i,k}+1},{\hat{l}}_{i,k}^{\left(m\right)}=\frac{{\hat{a}}_{i,k}^{\left(m\right)}-1}{{\hat{b}}_{i,k}^{\left(m\right)}}I\left({\hat{a}}_{i,k}^{\left(m\right)}>1\right),{\hat{\theta }}_{k,\cdot }^{\left(m\right)}=\sigma \left({\hat{\bar{\mu }}}_{k}^{\left(m\right)}\right).$$ (16)

We propose a heuristic procedure to select $\alpha_{0}^{\left(m\right)}$ . First, we apply the PoLNG model to data ***W*** ^(*m*)^, *m* = 1, 2, separately. With the estimated variational parameters in the Gamma distribution, we obtain the mean and variance of $l_{i,k,PoLNG}^{\left(m\right)}$ , denoted as $E\left(l_{i,k,PoLNG}^{\left(m\right)}\right)$ and $var\left(l_{i,k,PoLNG}^{\left(m\right)}\right)$ , respectively. Next, we fit a quantile regression with 90% quantile and no intercept term, with $var\left(l_{i,k,PoLNG}^{\left(m\right)}\right)$ being the dependent variable and $E^{2}\left(l_{i,k,PoLNG}^{\left(m\right)}\right)$ being the independent variable. Finally, $\alpha_{0}^{\left(m\right)}$ is computed as the reciprocal of the slope in the quantile regression.

The idea behind this heuristic procedure is based on Eq. 11, while the conditional mean and variance are approximated with the variational mean and variance. According to Eq. 11, there exists a linear relationship between $var\left(l_{i,k}^{\left(m\right)}|l_{i,k}\right)$ and $l_{i,k}^{2}$ with slope equal to $\frac{1}{\alpha_{0}^{\left(m\right)}}$ . By fitting ***W*** ^(*m*)^ with the PoLNG model, we are able to obtain the variational mean and variance, which approximate *l*_*i*,*k*_ and $var\left(l_{i,k}^{\left(m\right)}|l_{i,k}\right)$ , respectively. Considering the fact that the variance of the variational distributions is typically underestimated, we perform quantile regression with a high quantile rather than linear regression.

We also use the variational parameters obtained from fitting the PoLNG model to individual data modality as the warm start for the iPoLNG model. Because a large $\alpha_{0}^{\left(m\right)}$ indicates a small level of noise in data modality ***W*** ^(*m*)^, we define $\tilde{m}=\arg\max_{m}\alpha_{0}^{\left(m\right)}$ and use the variational parameters ${\bar{\mu }}_{k}^{\left(\tilde{m}\right)},{\bar{\Sigma }}_{k}^{\left(\tilde{m}\right)}$ obtained from the PoLNG model as the initial values for the variational parameters in the iPoLNG model. Also, to alleviate the effect of non-identifiability, we use $a_{i,k}^{\left(\tilde{m}\right)},b_{i,k}^{\left(\tilde{m}\right)}$ obtained from the PoLNG model as the initial values for the variational parameters for all *m* in the iPoLNG model. In the following analysis, the number of epochs is fixed to 3,000, the learning rate is set as 0.1, and the Adam optimizer is used in the SVI algorithm for both PoLNG and iPoLNG models.

## 3 Results

### 3.1 Real data analysis

To show that our model facilitates downstream analysis, iPoLNG is applied to several single-cell multiomics datasets, including one dataset generated from SHARE-seq, which measures gene expression and chromatin accessibility in the same single cells from a mouse brain, one dataset generated from Paired-Tag, which jointly profiles H3K27me3 histone modification and transcriptome in the same single cells from a mouse brain, and two cryopreserved human peripheral blood mononuclear (PBMC) datasets generated from 10X Genomics Single Cell Multiome ATAC + Gene Expression Sequencing.

For these datasets, we first filter out the low-quality cells that express in less than 500 genes in the gene expression data or in less than 200 regions in the epigenomic data. To select the informative features, we perform log-normalization with a scaling factor of 10,000 and select the top 5,000 highly variable genes and top 20,000 highly variable regions with selection.method = ‘‘vst” using R package Seurat (Stuart et al., 2019). The log normalizaion is merely used for selecting the highly variable features and the counts of the features are modeled by iPoLNG. Finally we take out the common cells in both data modalities as the input of the single cell multiomics data analysis.

#### 3.1.1 iPoLNG achieves good clustering performance on datasets from different technologies

We evaluate the clustering performance of iPoLNG on these datasets and compare our method with several existing methods designed for single-cell multiomics data integration, including scAI (Jin et al., 2020) and MOFA+ (Argelaguet et al., 2018; Argelaguet et al., 2020). scAI is implemented with the default parameters, and MOFA+ is implemented with the default parameters and two algorithms: mean-field variational inference (VI) using CPU and stochastic variational inference (SVI) with GPU acceleration. iPoLNG accepts raw count data as the input, while scAI and MOFA + accept the log-normalized data as the input. All these three methods can infer a low-dimensional representation of the data with a user-defined number of latent factors. We set the number of latent factors *K* = 50. After obtaining the cell by factor loading matrix, we perform Leiden clustering algorithm (Traag et al., 2019) with a binary search for the resolution parameter to cluster the data into the specific number of clusters. For datasets with given cell-type labels in the publications, the number of clusters is set as the number of the unique labels. The number of cluster is set as 8 for PBMC3k dataset and 19 for PBMC10k dataset.

For datasets with given cell-type labels, Adjusted Rand Index (ARI) (Hubert and Arabie, 1985) is computed to measure the accuracy of the clustering results. For PBMC datasets with unknown cell-type labels, Residual Average Gini Index (RAGI) score (Chen et al., 2019a) is computed based on canonical marker genes and housekeeping genes (see Supplementary Materials). A high RAGI score indicates a reasonable clustering result where the expression of marker genes is high in one or a few clusters, while the expression of housekeeping genes is broadly distributed across all the clusters. Considering the fact that the given cell-type labels in the original publications are also from some computational methods and can be wrong for some of the cells, we also compute the RAGI score. As Leiden clustering algorithm makes use of greedy search and leads to different clustering results with different initialization, we calculate the mean and standard error in 10 runs with different random seeds in the clustering step.

**FIGURE 2:**
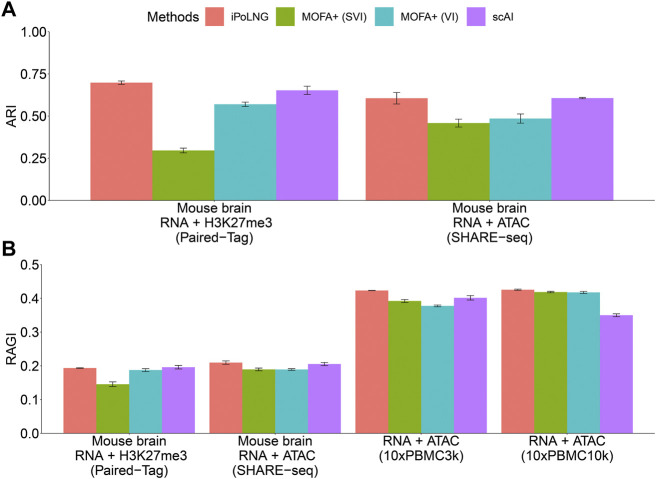
Clustering performance of iPoLNG, MOFA+ (SVI), MOFA+ (VI), and scAI on real data. **(A)** Comparison of ARI scores for Paired-Tag and SHARE-seq with given cell-type labels. **(B)** Comparison of RAGI scores for Paired-Tag, SHARE-seq, 10xPBMC3k, and 10xPBMC10k. The error bar represents the mean and standard error in 10 runs with different random seeds in the clustering step.

The clustering performance evaluated by ARI or RAGI is presented in Figure 2. In the Paired-Tag mouse brain dataset, iPoLNG achieves the highest ARI score (0.698), followed by scAI (0.653). In the SHARE-seq mouse brain dataset, iPoLNG reaches an ARI score of 0.606, which is comparable to the ARI score of 0.607 in scAI, although the clustering results of iPoLNG show a relatively high fluctuation. Neither VI nor SVI versions of MOFA + performs well in these two datasets. The clustering performance measured by RAGI also shows a trend similar to that measured by ARI for Paired-Tag and SHARE-seq mouse brain datasets. In 10xPBMC3k dataset, iPoLNG has the highest RAGI score (0.423). In 10xPBMC10k dataset, the RAGI score of iPoLNG is 0.426, slightly higher than that of MOFA+ (0.418 for SVI and 0.419 for VI), while scAI cannot perform as well as the other methods in this dataset.

In some applications, the cell structure revealed by different modalities can be different. We illustrate that iPoLNG is able to handle such scenarios by comparing the clustering performance of PoLNG (the simplified version of iPoLNG with just one data modality) with iPoLNG. In the Paired-Tag mouse brain dataset, the ARI score of running PoLNG for the single-cell RNA-seq data is 0.594, while the ARI score of running PoLNG for the single-cell histone modification data is very close to 0. In the SHARE-seq mouse brain dataset, the ARI score of running PoLNG for the single-cell RNA-seq data is 0.500, while the ARI score of running PoLNG for the single-cell ATAC-seq data is 0.02. The large difference in ARI between the two modalities indicates that the cell structure revealed by these modalities are different. When we integrate the information of both modalities using iPoLNG, the ARI score improves significantly compared to using RNA alone (from 0.594 to 0.698 in the Paired-Tag mouse brain dataset, and from 0.500 to 0.606 in the SHARE-seq mouse brain dataset).

#### 3.1.2 The factor loading matrices in iPoLNG provide rich biological insights

**FIGURE 3:**
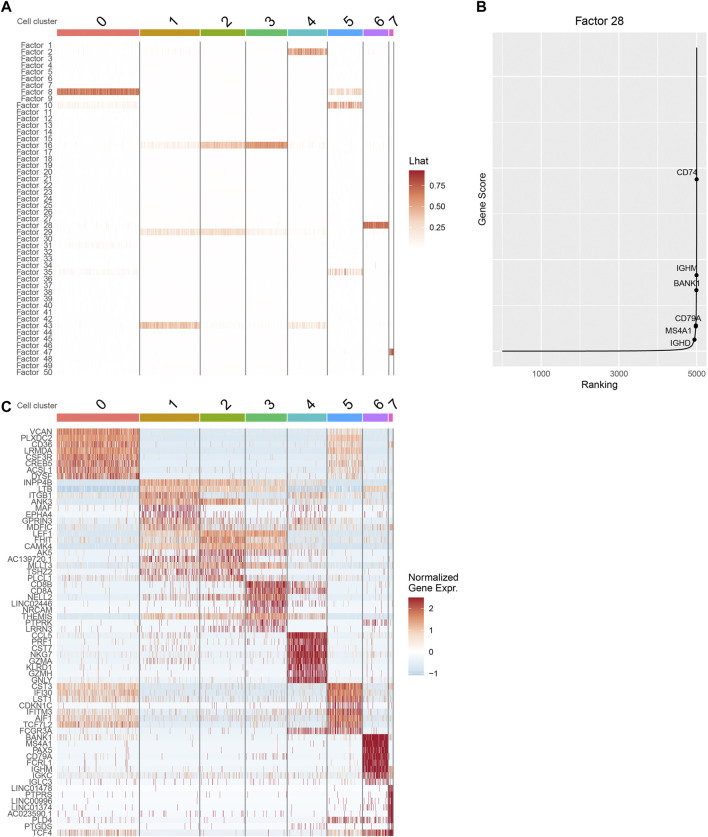
Downstream analysis with iPoLNG for 10xPBMC3k data. **(A)** The heatmap of the cell by factor loading matrix $\hat{L}$ . The major factor score of cluster 6 is allocated to factor 28. **(B)** Gene scores for ${\hat{\theta }}_{28,\cdot }^{\left(\text{RNA}\right)}$ , sorted in increasing order. The labelled marker genes of B cells tend to have high gene scores. **(C)** The heatmap for the top 8 cluster-specific differentially expressed genes for each cluster. This heatmap validates the similarity of cells within and across clusters. Cells in cluster 6 tend to have high gene expression values in the canonical marker genes of B cells.

We inspect the cell by factor loading matrix $\hat{L}$ inferred by iPoLNG for the 10xPBMC3k dataset (Figure 3A) and the heatmap for the top 8 differentially expressed genes for each cluster (Figure 3C). The differentially expressed genes are found by the FindAllMarkers() function using R package Seurat. Similarity in the factor loading matrix tends to be consistent with the similarity in the heatmap of marker genes: for example, clusters 1, 2 and 3 tend to have high factor scores for factors 16 and 29 (Figure 3A), and their expression pattern for the marker genes tend to be more similar to each other (Figure 3C).

Next, we focus on cluster 6, whose major factor score is allocated to factor 28. From the heatmap of gene expression, we find that cells in cluster 6 tend to have high gene expression values in canonical marker genes of B cells, including BANK1, MS4A1, CD79A, and IGHM (Figure 3C). By plotting ${\hat{\theta }}_{28,\cdot }^{\left(\text{RNA}\right)}$ according to the ranking of gene factor scores (Figure 3B), the canonical marker genes of B cells also tend to have large gene factor scores, which is consistent with the conclusion from the heatmap of gene expression.

We also perform gene ontology (GO) enrichment analysis using the feature by factor loading matrices ${\hat{\Theta }}^{\left(\text{RNA}\right)}$ and ${\hat{\Theta }}^{\left(\text{ATAC}\right)}$ . We still focus on cluster 6 and factor 28 in 10xPBMC3k data. More specifically, we select the top 200 genes with large factor scores in ${\hat{\theta }}_{28,\cdot }^{\left(\text{RNA}\right)}$ as the input of Metascape (Zhou et al., 2019), and the top 1,000 regions with large factor scores in ${\hat{\theta }}_{28,\cdot }^{\left(\text{ATAC}\right)}$ as the input of Genomic Regions Enrichment of Annotations Tool (GREAT) (McLean et al., 2010). The results for Metascape and GREAT are presented in Supplementary Files S1, S2, respectively. The enriched biological processes and pathways with highly significant p-values include immune response, regulation of lymphocyte activation and pathways that are highly related to B cells. In conclusion, the GO enrichment analysis agrees well with the previous analysis of marker genes on cluster 6.

#### 3.1.3 iPoLNG is able to handle partial information in the input

In some applications, we have one dataset that have multiple modalities, but the other dataset that only measures one of the modalities, and we expect that the dataset with only one modality can be jointly trained with the multi-modal dataset so that it can borrow some information from the multi-modal dataset. iPoLNG is able to handle such partial information in the input by setting the unobserved count as 0, which is mathematically equivalent to not including the unobserved data in the likelihood function of the data.

**FIGURE 4:**
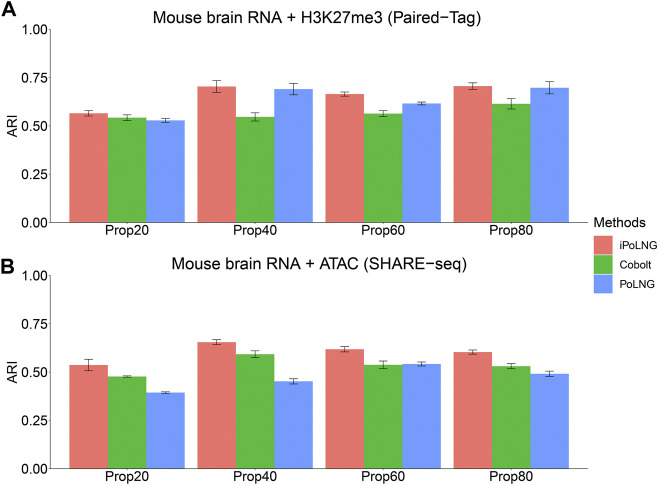
Clustering performance of iPoLNG, Cobolt and PoLNG on the partially masked dataset for **(A)** Paired-Tag mouse brain RNA + H3K27me3 and **(B)** SHARE-seq mouse brain RNA + ATAC. We set the percentage of masked cells to be 20%, 40%, 60% and 80% of all the cells.

We design a new experiment to illustrate the power of iPoLNG to handle partial information and compare the result with Cobolt (Gong et al., 2021), which also enables integrating single-modality dataset with multi-modal dataset. First, for the epigenomic data modality ***W*** ^(2)^, we randomly mask the data matrix for a certain percentage of the cells by setting the observed count as 0, i.e., $W^{\left(2\right)}={\left(W_{\mathrm{unmasked}}^{{\left(2\right)}^{\text{T}}},W_{\mathrm{masked}}^{{\left(2\right)}^{\text{T}}}\right)}^{\text{T}}$ and $W_{\mathrm{masked}}^{\left(2\right)}=O$ is a zero matrix. Correspondingly, we denote the transcriptomic data modality $W^{\left(1\right)}={\left(W_{\mathrm{unmasked}}^{{\left(1\right)}^{\text{T}}},W_{\mathrm{masked}}^{{\left(1\right)}^{\text{T}}}\right)}^{\text{T}}$ , where $W_{\mathrm{masked}}^{{\left(1\right)}^{\text{T}}}$ represents the transcriptomic data for the cells in which the epigenomic modality is masked. Next, we apply PoLNG to the transcriptomic data modality of these masked cells, $W_{\mathrm{masked}}^{{\left(1\right)}^{\text{T}}}$ . We apply iPoLNG and Cobolt to ${\left(W_{\mathrm{unmasked}}^{{\left(1\right)}^{\text{T}}},W_{\mathrm{masked}}^{{\left(1\right)}^{\text{T}}}\right)}^{\text{T}}$ and ${\left(W_{\mathrm{unmasked}}^{{\left(2\right)}^{\text{T}}},O^{\text{T}}\right)}^{\text{T}}$ , where both transcriptomic and epigenomic data are observed for unmasked cells, and only transcriptomic data is observed for masked cells. Finally, we perform Leiden clustering on the low-dimensional embeddings of the masked cells in PoLNG, iPoLNG and Cobolt, respectively, and measure the clustering performance by computing their ARI scores. We set the percentage of masked cells to be 20%, 40%, 60% and 80% of all the cells, and the results for the Paired-Tag mouse brain dataset and the SHARE-seq mouse brain dataset are presented in Figure 4. The clustering performance of iPoLNG is better than that of Cobolt and PoLNG under all settings, showing the power of iPoLNG to enable a dataset with single modality to borrow information from a larger dataset with two modalities.

### 3.2 Model validation and comparison using simulated data

We next perform simulation study to demonstrate the effectiveness of our proposed method.

To generate simulated data, we first fit the iPoLNG model with *K* = 50 to one dataset from Paired-Tag, where ***W*** ^(1)^ is the transcriptome data and ***W*** ^(2)^ is the H3K27me3 histone modification data of a mouse brain, and obtain the hyperparameters $\alpha_{0}^{\left(1\right)},\alpha_{0}^{\left(2\right)}$ and the fitted variational parameters $\hat{L},{\hat{\Theta }}^{\left(1\right)},{\hat{\Theta }}^{\left(2\right)}$ . Next, we take a subset of this dataset to obtain all cells in the following five cell types “HC_ExNeu_CA1” (403 cells), “FC_ExNeu_PT” (219 cells), “HC_ExNeu_DG” (396 cells), “BR_InNeu_CGE” (169 cells), “HC_ExNeu_CA23” (440 cells), calculate the column mean of $\hat{L}$ within each cluster to obtain “cluster centers” ${\bar{l}}_{1,\cdot },{\bar{l}}_{2,\cdot },{\bar{l}}_{3,\cdot },{\bar{l}}_{4,\cdot },{\bar{l}}_{5,\cdot }$ . In the simulated data, we assume that there are 5 clusters and the cells in the *i*th cluster are generated from ${\bar{l}}_{i,\cdot }$ . More specifically, we utilize the hyperparameters $\alpha_{0}^{\left(1\right)},\alpha_{0}^{\left(2\right)}$ , cluster centers ${\bar{l}}_{1,\cdot },{\bar{l}}_{2,\cdot },{\bar{l}}_{3,\cdot },{\bar{l}}_{4,\cdot },{\bar{l}}_{5,\cdot }$ , the fitted variational parameters ${\hat{\Theta }}^{\left(1\right)},{\hat{\Theta }}^{\left(2\right)}$ and the sequencing depth obtained from all 1,627 cells in the 5 clusters to generate simulated data according to our generative model (10) (See Supplementary File S3 for the value of fitted variational parameters and the sequencing depths for the cells.) In order to evaluate the performance of our algorithm for data under different levels of noise, we divide the sequencing depth in the transcriptomic data by 1,2,5,10 respectively to generate simulated datasets with 4 different levels of noise. We expect that datasets with small sequecing depths tend to have low UMI counts, thus high sparsity and a high level of noise. For each setting, 5 datasets are generated with different seeds.

**FIGURE 5:**
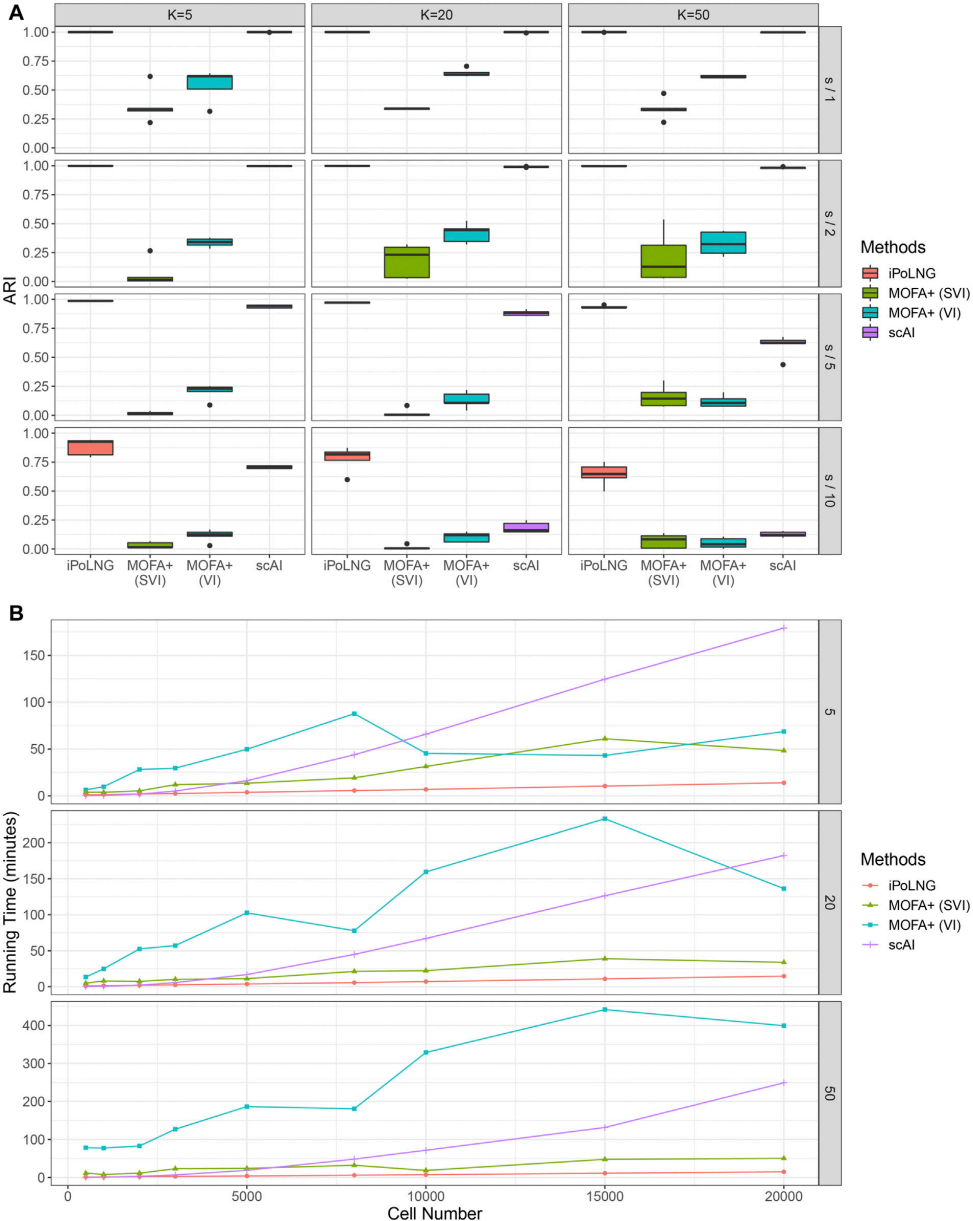
Comparison of clustering performance and running time for simulated data. **(A)** ARI scores for *K* = 5, 20, 50 and the level of noise is adjusted by dividing the sequencing depth by 1, 2, 5, and 10. The boxplot represents the ARI scores for 5 simulated datasets under the same setting. **(B)** Computational time for iPoLNG, MOFA+ and scAI. MOFA+ (VI) and scAI were run on a server with Intel Xeon Gold 6246R CPU and 120 GB RAM. iPoLNG and MOFA+ (SVI) were run on a server with NVIDIA Tesla V100 GPU and 80 GB RAM.

We again evaluate the clustering performance of iPoLNG and compare our method with scAI and MOFA+. We varied the number of factors *K* as 5, 20, 50 for all methods. The boxplots of ARI values for the simulated datasets are presented in Figure 5A. When the level of noise is low (sequencing depth divided by 1 or 2), both iPoLNG and scAI can reach ARI values of nearly 1, which suggests that they can accurately recover the cell types in the simulated data. As the level of noise increases, the performance of all methods becomes worse as expected, but iPoLNG still remains the best method among all settings. We also note that iPoLNG is robust to the choice of the number of factors. When *K* is larger than 5, i.e. the number of cell types, the clustering performance of iPoLNG does not decrease significantly under small or moderate levels of noise.

We also evaluate the running time of the methods (Figure 5B). Cells are sampled with replacement from the preprocessed 10xPBMC3k dataset to generate simulated data with different numbers of cells. With GPU acceleration, the running time of iPoLNG for the simulated dataset with 20,000 cells is 13.9 min for *K* = 5, 14.7 min for *K* = 20 and 14.9 min for *K* = 50, which remains the smallest among all the methods under the same setting. MOFA+ (SVI) is the second fastest method, but its running time is 2–6 times the running time of iPoLNG. The slight change of running time across *K* also illustrates iPoLNG’s running time is robust to the number of factors *K*. By contrast, the running time of MOFA+ and scAI can be significantly affected by the number of factors.

## 4 Discussion

Single-cell multiomics technologies generate datasets with multi-modal measurements from the same set of cells, thus posing significant challenges for integrating and characterizing multiple types of measurements in a biologically meaningful way. The single-cell data is high-dimensional yet intrinsically sparse, and different layers of single-cell multiomics data usually exhibit different levels of noise.

In this study, we introduced iPoLNG, an unsupervised method for integrating single-cell multiomics data to dissect the cellular heterogeneity from multiple data modalities. From a biological perspective, iPoLNG infers two kinds of low-dimensional representations of the high-dimensional single-cell multiomics data: one cell by factor loading matrix and two feature by factor loading matrices. The cell by factor loading matrix can identify distinct cell types and improve clustering accuracy compared to other models that reconstruct the latent space of cells, and the feature by factor loading matrices can characterize cell-type specific markers and facilitate gene ontology (GO) enrichment analysis. From a technical perspective, iPoLNG presents several advantages. First, it directly models the unique molecular identifiers (UMIs) of single-cell multiomics data and takes into account the sequencing depths of cells, which suggests the discrete counts without any normalization procedure can directly serve as the input of the model. Second, as a scalable algorithm, stochastic variational inference with GPU acceleration in iPoLNG potentially enables the computation of large-scale single-cell datasets with a considerably high speed. Third, the hyperparameters that control the levels of noise across different data modalities in iPoLNG are automatically learned by fitting the PoLNG model to individual data modality, which saves the efforts to tune these hyperparameters.

iPoLNG also exhibits some limitations. First, modelling the discrete counts directly suggests that it lacks the flexibility to fit continuous data. Second, this method is tailored specifically for multi-modal measurements from the same sample space, contrasting with some other methods (Stuart et al., 2019; Welch et al., 2019) that aim at integrating cells on the same feature space. Third, iPoLNG assumes independence between features by a diagonal covariance matrix in the Logit-Normal distribution, but genomic features are known to show interaction *via* gene regulatory networks (Duren et al., 2017; Colomé-Tatché and Theis, 2018; Delgado and Gómez-Vela, 2019).

We speculate the future direction of iPoLNG as follows. We may incorporate the idea of Deep Exponential Families (Ranganath et al., 2015) to model the complex biological structures by adding additional layers for the latent factors. The model may also be extended to analyze spatial epigenome-transcriptome co-profiling data by modelling the information of spatial coordinates with links (Chang and Blei, 2009). Additionally, the model may be extended to incorporate the regulatory links between transcriptome and epigenome (Colomé-Tatché and Theis, 2018).

## Data Availability

Publicly available datasets were analyzed in this study. This data can be found here: https://www.ncbi.nlm.nih.gov/geo/query/acc.cgi?acc=GSE140203 https://www.ncbi.nlm.nih.gov/geo/query/acc.cgi?acc=GSE152020 https://www.10xgenomics.com/resources/datasets/pbmc-from-a-healthy-donor-granulocytes-removed-through-cell-sorting-10-k-1-standard-2-0-0. The processed data can also be accessed from GitHub repository: iPoLNG_source. https://github.com/cuhklinlab/iPoLNG_source. iPoLNG is implemented in Python, and it is freely available under the LGPL-3.0 license on GitHub (https://github.com/cuhklinlab/iPoLNG).
