# Supplementary material for: iPoLNG—An unsupervised model for the integrative analysis of single-cell multiomics data
Source: Front Genet. 2023 Feb 7;14:998504. doi: 10.3389/fgene.2023.998504 (PMC9972291; doi:10.3389/fgene.2023.998504)
Supplement: Supplementary file 1 [file DataSheet2.PDF]

Supplementary Material

1 SUPPLEMENTARY DATA

List of housekeeping genes used for computing Residual Average Gini Index (RAGI): ACTB, ALDOA, GAPDH, PGK1, LDHA, RPS27A, RPL19, RPL11, NONO, ARHGDIA, RPL32, RPS18, HSPCB, C1ORF43, CHMP2A, EMC7, GPI, PSMB2, PSMB4, RAB7A, REEP5, SNRPD3, VCP, VPS29.

List of marker genes used in 10xPBMC3k and 10xPBMC10k datasets for computing Residual Average Gini Index (RAGI): CD209, ENG, FOXP3, CD34, BATF3, S100A12, THBD, CD3D, THY1, CD8A, CD8B, CD14, PROM1, IL2RA, FCGR3A, IL3RA, FCGR1A, CD19, IL7R, CD79A, MS4A1, NCAM1, CD3E, CD3G, KIT, CD1C, CD68, CD4.

List of marker genes used in Paired-Tag mouse brain dataset for computing Residual Average Gini Index (RAGI): Dnah12, Cfap299, Flt1, Slco1a4, Inpp5d, Hexb, Slc1a3, Atp1a2, Prr5l, Plp1, Rnf220, Lhfpl3, Cacng4, Erbb4, Kcnc2, Reln, Grin3a, Adarb2, Fam19a2, Ahcyl2, Gm32647, Shisa6, Epha6, Galnt14, Tshz2, Fam19a1, Il1rapl2, Cdh12, Rgs6, Lingo2, Zfp804b, Pex5l, Gm26883, Cdh18, Galntl6, Spag16, Cfap43, Wdr49, Ebf1, Mecom, Ptprb, Tgfbr1, Zfhx3, Apbb1ip, Gpc5, Plpp3, Slc1a2, Igsf8, St18, Mog, Mag, Mbp, Vcan, Tnr, 6030443J06Rik, Nxph1, 6330411D24Rik, Kcnmb2, Gm45341, Gm45455, Grip1, Trpm3, Rfx3, Dgkh, Hs3st4, Ryr3, Grik4, Cpne7, Hs6st3, Sdk1, Foxp2, Garnl3, Grm8, Vwc2l, Olfm3, Grik1, Gm2164, Gm28928, Sgcz, Prr16, Chrm3, Pdzrn3, Kcnq5, Unc5d, Car10, Pcdh15, Nrg1.

List of marker genes used in SHARE-seq mouse brain dataset for computing Residual Average Gini Index (RAGI): Meg3, Rbfox3, Slc17a7, Gad1, Gad2, Etv1, Gja1, Aqp4, Mbp, Mog, Pdgfra, Dnah12, Flt1, Slco1a4, Inpp5d, Hexb, Slc1a3, Atp1a2, Slc1a2, Gpc5, Prr5l, Plp1, Rnf220, Lhfpl3, Cacng4, Erbb4, Kcnc2, Reln, Grin3a, Adarb2, Fam19a2, Ahcyl2, Shisa6, Epha6, Galnt14, Hs3st4, Tshz2, Fam19a1, Il1rapl2, Cdh12, Rgs6, Lingo2, Prr16, Zfp804b, Pex5l, Cdh18, Gm28928, Galntl6.
